# Influence of Trace Mineral Sources and Levels on Growth Performance, Carcass Traits, Bone Characteristics, Oxidative Stress, and Immunity of Broiler

**DOI:** 10.3390/ani15152287

**Published:** 2025-08-05

**Authors:** Tassanee Trairatapiwan, Rachakris Lertpatarakomol, Sucheera Chotikatum, Achara Lukkananukool, Jamlong Mitchaothai

**Affiliations:** 1 Faculty of Veterinary Medicine, Mahanakorn University of Technology (MUT), Bangkok 10530, Thailand; tassanee@mut.ac.th (T.T.); sucheera@mut.ac.th (S.C.); 2 Department of Animal Production Technology and Fisheries, School of Agricultural Technology, King Mongkut’s Institute of Technology Ladkrabang (KMITL), Bangkok 10520, Thailand; achara.lu@kmitl.ac.th; 3 Office of Administrative Interdisciplinary Program on Agricultural Technology, School of Agricultural Technology, King Mongkut’s Institute of Technology Ladkrabang (KMITL), Bangkok 10520, Thailand; jamlong.mi@kmitl.ac.th

**Keywords:** inorganic minerals, organic mineral, trace mineral, broiler

## Abstract

This study investigated the effects of reducing organic trace minerals below commercial inclusion levels and compared them with both low-dose and commercial levels of inorganic trace minerals, focusing on growth performance, carcass traits, tibia characteristics, oxidative stress (superoxide dismutase [SOD] and malondialdehyde [MDA]), and immune response (serum IgG) in broilers. A total of 384 one-day-old Ross 308 chicks were randomly assigned to three dietary treatments: (1) commercial-level inorganic trace minerals (ILI; Zn 100 ppm; Cu 15 ppm; Fe 100 ppm; Mn 80 ppm; Se 0.2 ppm; I 3 ppm); (2) low-level organic trace minerals (LLO; Zn 30 ppm; Cu 4 ppm; Fe 11 ppm; Mn 30 ppm; Se 0.225 ppm; I 3 ppm), and (3) low-level inorganic trace minerals (LLI; Zn 30 ppm; Cu 4 ppm; Fe 11 ppm; Mn 30 ppm; Se 0.2 ppm; I 3 ppm). Each treatment consisted of eight replicates with 16 birds per replicate, and diets were provided in two phases: starter (days 1–21) and grower (days 22–35). The results showed that the LLO group demonstrated a significantly improved feed conversion ratio (FCR) during the starter phase, 2.4% better than that of the ILI and LLI groups (*p* = 0.02). Additionally, filet and thigh muscle yields in the LLO group were higher by 11.9% (*p* = 0.03) and 13.9% (*p* = 0.02), respectively, compared to the ILI group. Other carcass traits, as well as pH and drip loss, were not significantly affected. However, tibia breaking strength at day 35 was 15.1% lower in the LLO group compared to the ILI group (*p* = 0.02). No significant differences were observed in oxidative stress markers or IgG levels among groups. This study demonstrated that reducing the inclusion level of inorganic trace minerals did not negatively affect broiler growth performance, whereas supplementation with low levels of organic trace minerals improved both growth performance and carcass quality.

## Simple Summary

Trace minerals (zinc, copper, iron, manganese, selenium, and iodine) are essential for optimal growth and health in broiler chickens. However, they are often added to poultry diets at high levels in the form of low-cost inorganic compounds that are poorly absorbed. This study investigated whether low levels of highly bioavailable organic trace minerals could support broiler health and performance while reducing environmental mineral excretion. Broilers were assigned to diets containing either commercial levels of inorganic trace minerals, low levels of organic trace minerals, or low levels of inorganic trace minerals. Birds receiving low levels of organic trace minerals exhibited improved feed conversion ratios during the starter phase and showed increased filet and thigh muscle yields. Bone development, oxidative stress markers, and humoral immune responses were not affected by either the source or inclusion level of dietary trace minerals. Notably, reducing the inclusion level of organic trace minerals did not compromise broiler growth or health, indicating more efficient mineral utilization. Similarly, reducing inorganic trace mineral supplementation did not impair performance, suggesting that current commercial inclusion levels may exceed physiological requirements. These findings suggest that incorporating low levels of organic trace minerals in broiler diets can sustain productivity while promoting more sustainable and environmentally responsible poultry production practices.

## 1. Introduction

Trace minerals, though required in small quantities, are essential for maintaining optimal physiological, enzymatic, and immune functions in animals. Elements such as zinc (Zn), copper (Cu), manganese (Mn), selenium (Se), iron (Fe), and iodine (I) are critical cofactors for a variety of biological processes, including growth, bone development, oxidative balance, and immunological responses [1,2,3]. In commercial poultry production, achieving precise trace mineral nutrition is vital to support performance, health, and product quality [4,5,6].

Conventional broiler diets often rely on inorganic trace mineral (ITMs) sources, such as sulfates, oxides, and carbonates, due to their low cost and ease of formulation [5]. However, these forms are generally characterized by poor bioavailability, leading to their over-supplementation to meet metabolic needs [5,6]. In addition, Thailand’s hot and humid climate increases the risk of mycotoxin contamination in feed ingredients [7,8]. Mycotoxin contamination can induce a range of physiological disturbances, including oxidative stress and immunosuppression, thereby increasing the requirement for antioxidant-related trace minerals, which serve as essential cofactors for enzymes like glutathione peroxidase and superoxide dismutase [7,9]. In addition, mycotoxins compromise both innate and adaptive immune responses, necessitating elevated levels of trace minerals to support immune function and enhance disease resistance [8]. To mitigate these risks and ensure adequate nutrient supply under such challenging conditions, animal feed manufacturers often supplement diets with vitamins and trace minerals at levels exceeding the recommendations of research institutions by 100 to 500% as a safety margin [5,10]. This excessive use contributes to mineral antagonism, oxidative stress, and elevated excretion rates, thereby posing risks to animal health and environmental sustainability [5,6,10].

Organic trace minerals (OTMs), which involve chelation or complexation with amino acids, peptides, or organic acids, offer a more bioavailable and stable alternative to traditional inorganic forms [11,12,13]. Numerous studies have demonstrated that OTMs can maintain or even enhance performance indicators such as body weight gain and feed conversion ratio, often at lower inclusion levels than their inorganic counterparts [11,14]. Additionally, OTMs have been associated with improvements in carcass characteristics, including increased breast meat yield and reduced abdominal fat [12,14]. Benefits also extend to meat quality, with reports indicating reduced drip loss, decreased lipid peroxidation, and improved tenderness [12,15]. Importantly, the superior absorption and retention of OTMs contribute to lower mineral excretion, representing a nutritional strategy that enhances broiler productivity while reducing environmental impact [11,14,16]. Furthermore, OTMs align with the principles of precision nutrition and sustainable animal production by enabling reduced dietary inclusion levels without compromising performance outcomes [11,12,14].

While numerous studies have compared OTMs and ITMs, few have investigated the effects of reducing both sources below current commercial inclusion levels. The present study addresses this research gap by simultaneously evaluating the impact of reduced-dose OTMs and ITMs supplementation in broilers. Unlike previous research, this study was specifically designed to test the hypothesis that OTMs, due to their superior bioavailability, can be used at lower inclusion levels without compromising performance or health outcomes. Additionally, it aimed to assess the effects of lowering ITMs below standard commercial levels. This dual comparison provides valuable insight into the potential for optimizing trace mineral utilization in broiler diets while maintaining productivity and minimizing environmental impact, thereby aligning with the principles of precision nutrition and sustainable poultry production. Therefore, this study aims to evaluate the effects of trace mineral sources and levels on growth performance, carcass traits, bone characteristics, oxidative stress, and immune response of broiler chicken.

## 2. Materials and Methods

### 2.1. Animal Cares

The present experiment was reviewed and approved by the Institutional Animal Care and Use Committee of Mahanakorn University of Technology (ACUC-MUT-2024/009).

### 2.2. Animals, Diets, and Experimental Design

This study was conducted at Faculty of Veterinary Medicine, Mahanakorn University of Technology. All chicks were raised in a 6 × 10 m^2^ evaporative cooling system house. A total of 384 Ross 308, one-day-old broiler chickens were randomly distributed into twenty-four 1 × 1.2 cm^2^ metal mesh pallet cages with rice husk as litter material cages as completely randomized experimental design (CRD) into 3 treatments with 8 replicates of 16 birds in each. The experimental treatments consisted of Control group: commercial levels of ITMs (ILI; Zn 100 ppm; Cu 15 ppm; Fe 100 ppm; Mn 80 ppm; Se 0.2 ppm; I 3 ppm), replace trace minerals with low levels of OTMs (LLO; Zn 30 ppm; Cu 4 ppm; Fe 11 ppm; Mn 30 ppm; Se 0.225 ppm; I 3 ppm), and low levels of ITMs (LLI; Zn 30 ppm; Cu 4 ppm; Fe 11 ppm; Mn 30 ppm; Se 0.2 ppm; I 3 ppm).

The feeding program was divided into two phases: a starter diet (days 1–21) formulated to contain 22% crude protein (CP) and 3100 kcal of metabolizable energy (ME)/kg, and a grower diet (days 22–35) formulated to contain 20% CP and 3150 kcal ME/kg. These dietary formulations were adapted from the recommendations of the NRC (1994) [17] and the Ross 308 nutrient specification guidelines (Aviagen, Huntsville, AL, USA). All diets were pellet feed. All birds were given ad libitum access to feed and drinking water. The composition of the basal diets showed in Table 1. Routine medication, vaccination, and husbandry practices were administered. Growth performance data were collected and analyzed for accumulated periods of 1 to 21, 22 to 35, and 1 to 35 days of age. The body weight gain (BWG), feed intake (FI), and feed conversion ratio (FCR) of all treatments were analyzed.

### 2.3. Carcass Characteristics and Meat Quality

At 35 days of age, carcass yield was determined using two birds per replicate pen. Birds were subjected to a 6 h pre-slaughter fasting period, individually identified, and manually slaughtered. The slaughter process included bleeding, scalding, and feather removal, after which carcasses were stored overnight at 2 ± 2 °C. For carcass yield calculation, the cold carcass weight including feet, head, and offal was recorded and expressed as a percentage of the live body weight measured prior to slaughter. Yields of primal cuts (including bone and skin) were calculated based on the live weight and included the entire head and neck, breast, filet, thigh, drumstick, wing, and ribs. The heart, gizzard, and liver were also weighed and expressed relative to live body weight.

The ultimate pH of the breast muscle was measured 24 h post-mortem using a pH meter (ECPH70042GS, Eutech Instruments Pte Ltd., Singapore City, Singapore), which was calibrated with standard buffers of pH 4.0 and 6.9. Approximately 10 g of ground breast meat was mixed with 100 mL of distilled water, blended at high speed for 30 s, and immediately transferred to a 250 mL glass beaker. The electrode was then inserted to record pH. For drip loss, breast muscle samples were weighed, sealed in zip-lock plastic bags, stored at 2 ± 2 °C for 24 h, and reweighed to calculate drip loss as a percentage of initial weight.

### 2.4. Bone Characteristics

Two samples of the right tibia from each broiler chicken were collected to evaluate fresh weight. The samples were then dried at room temperature for 24 h and weighed using a digital scale. Bone dimensions were measured using a digital Vernier caliper, including the length from the proximal to the distal end and the width at the narrowest and widest points. Each bone was sectioned longitudinally to measure the diameter of the diaphysis and the medullary canal. The samples were further dried in a hot air oven at 138 °C until a constant weight was achieved, then cooled in a desiccator before weighing. Bone ash content was determined by incinerating the samples at 600 °C for 4–6 h, following the AOAC (2012) method [18].

Two samples of the left tibia from each broiler were used to measure bone hardness using a hardness tester (model LR5K, Llyod Instruments Ltd., Hampshire, UK), equipped with a 500 N load cell. The test was performed at a distance of 40 mm from the platform with an indenter speed of 5 mm per minute.

### 2.5. Determinations of Oxidative Stress and Humeral Immunity

In this study, oxidative stress and humoral immunity in broiler chickens were assessed by measuring superoxide dismutase (SOD) activity, malondialdehyde (MDA) concentration, and total serum IgG antibody levels. At days 21 and 35, blood samples were collected from two birds of each replicate via wing vein puncture and centrifuged at 3000 rpm for 15 min to obtain serum. All oxidative stress parameters (SOD and MDA) and IgG concentrations were measured using serum samples. The activity of SOD (expressed as inhibition rate %) was determined using the S311 SOD Assay Kit—WST (Dojindo Laboratories, Tokyo, Japan), while MDA concentration was measured using the M496 MDA Assay Kit (Dojindo Laboratories, Tokyo, Japan). Total IgG levels were quantified using the MBS260043 Chicken IgG ELISA Kit (MyBioSource, Inc., San Diego, CA, USA).

### 2.6. Statistical Analysis

All data were analyzed using one-way analysis of variance (ANOVA) in SPSS version 17.0 (SPSS Inc., Chicago, IL, USA). When a significant treatment effect was observed (*p* < 0.05), means were separated using Duncan’s New Multiple Range Test.

## 3. Results

### 3.1. Growth Performance

The effects of trace mineral sources and levels on broiler growth performance are presented in Table 2. During the starter phase (1–21 days), the LLO group demonstrated a significantly improved feed conversion ratio (FCR) during the starter phase, being 2.4% better than that of the ILI and LLI groups (*p* = 0.02). However, no significant differences (*p* > 0.05) were observed among treatments in FI, BWG, or FCR over the entire rearing period (1–35 days). Additionally, there were no significant performance differences between the ILI and LLI groups (*p* > 0.05).

### 3.2. Carcass Characteristics and Meat Quality

As shown in Table 3, filet and thigh muscle yields in the LLO group were higher by 11.9% (*p* = 0.03) and 13.9% (*p* = 0.02), respectively, compared to the ILI group. However, no significant differences (*p* > 0.05) were observed among treatments for the head and neck, wing, breast, drumstick, leg and feet, ribs, internal organs, pH, or drip loss.

### 3.3. Bone Characteristics

Table 4 showed that tibia length, diameter, weight, and ash content did not differ significantly (*p* > 0.05) among treatments. However, tibia breaking strength at day 35 was 15.1% lower in the LLO group compared to the ILI group (*p* = 0.02).

### 3.4. Oxidative Stress and Immunity

According to Table 5, trace mineral source and level had no significant effect (*p* > 0.05) on oxidative stress markers (SOD and MDA) or serum IgG antibody levels.

## 4. Discussion

### 4.1. Growth Performance

In a study examining the effects of various trace mineral sources on broiler performance, it was found that during the starter phase (1–21 days), grower phase (22–35 days), and the entire rearing period (1–35 days), using OTMs at levels equivalent to the reduced levels of ITMs commonly used in commercial broiler diets (LLO) did not negatively impact FI, BWG, FCR, or mortality. In fact, broilers fed OTMs at reduced levels during the starter phase exhibited improved FCR. These findings are consistent with those of Nollet et al. [19], who reported that supplementing OTMs at levels lower than current inorganic recommendations did not adversely affect broiler growth performance across all rearing phases and showed a trend toward improved FCR in the early phase (1–14 days) compared to ITMs (*p* = 0.06). This suggests that OTMs can be effectively used at lower inclusion rates in broiler feed formulations.

The present study also evaluated the effect of reducing ITM levels to those commonly used in commercial diets (LLI). When compared to OTMs at the same reduced level (LLO), broilers receiving OTMs demonstrated better FCR during the early phase, further supporting the findings of Nollet et al. [19]. While OTMs improved early growth efficiency, they did not influence performance during the grower phase or over the entire period, differing from the findings of M’Sadeq et al. [20], who observed improved FCR at the end of the rearing period when using OTMs, even at reduced levels. However, the current findings are in line with those of Franklin et al. [11] and Núñez et al. [21], who reported no significant differences in broiler performance when equal levels of OTMs and ITMs were used throughout the growth cycle.

These inconsistencies suggest that the effects of trace mineral sources on growth performance may vary. This aligns with the conclusion of Świątkiewicz et al. [22], who noted that the response to OTMs in poultry diets is not always consistent, particularly regarding growth performance. Variability may arise from several factors, including the type of trace minerals used (organic vs. inorganic), mineral inclusion levels, feed formulation, and animal-related factors.

Additionally, reducing ITMs to the lower levels typical of commercial diets (LLI) did not negatively affect growth performance at any stage, indicating that current industry recommendations may exceed actual broiler requirements. This is likely due to the inclusion of broad safety margins in commercial formulations. These findings support the possibility of reducing ITM inclusion rates without compromising growth performance, in agreement with Franklin et al. [13], who found that reducing dietary inorganic Zn, Mn, and Cu had no negative effect on broiler performance, as dietary supply remained sufficient. Furthermore, mineral levels in commercial premixes are often much higher than the animals’ requirements, leading to excess excretion and environmental pollution [12,16].

### 4.2. Carcass Characteristics and Meat Quality

In this study, filet and thigh percentages were significantly higher (*p* < 0.05) in broilers receiving low levels of OTMs (LLO) compared to those receiving commercial levels of ITMs (ILI), suggesting enhanced muscle development associated with organic mineral supplementation. Organic minerals, often in the form of chelates or proteinates, are bound to organic ligands such as amino acids, peptides, or proteins. This binding alters the physicochemical properties of the minerals, improving their stability and bioavailability within the gastrointestinal tract. The neutral or slightly positive net charge of these complexes reduces antagonistic interactions with other dietary components and prevents the formation of insoluble complexes, which are common with inorganic minerals [1,5]. As a result, OTMs are absorbed more efficiently through specific amino acid or peptide transport systems, bypassing the less selective and competitive pathways used by inorganic minerals [23,24]. In addition, previous study concluded that the use of organic Zn did not influence the content of basic chemical components or the pH of broiler meat [25].

The superior bioavailability of OTMs can enhance metabolic functions, including protein synthesis, enzyme activity, and antioxidant defense, all of which are critical for muscle accretion and tissue development [20,26]. Specifically, trace elements such as Zn and Mn play vital roles in protein metabolism, collagen synthesis, and cellular proliferation, which may explain the observed increases in filet and thigh yields [9,13,27].

Moreover, several studies have demonstrated that reduced inclusion levels of OTMs, compared to traditional inorganic sources, can maintain or even improve performance and carcass traits due to their higher utilization efficiency [12,18]. Additionally, previous studies have demonstrated that organic zinc supplementation does not influence the basic chemical composition or pH of broiler meat [24]. This not only promotes better nutrient conversion into lean tissue but also supports precision nutrition by minimizing excess mineral excretion and reducing environmental impact.

### 4.3. Bone Characteristics

The current study revealed that most measured bone parameters, including tibia length, diameter, ash content, and weight, were not significantly affected by the different treatments (*p* > 0.05). These findings align with those of M’Sadeq et al. [20], who reported that reducing OTMs to levels below those recommended for ITMs did not negatively impact tibia size, ash percentage, or breaking strength in 38-day-old broilers. The lack of substantial variation suggests that, even at reduced inclusion levels, trace minerals—whether organic or inorganic may meet the mineral requirements necessary for bone development in modern broiler genotypes [4].

Specifically, using OTMs at levels equivalent to reduced ITM inclusion (LLO) did not affect bone size, ash content, or strength at both 21 and 35 days of age. These results further support the findings of M’Sadeq et al. [20], indicating that OTMs can be used at lower levels without compromising bone development.

The limited impact of trace mineral form and concentration on bone characteristics likely stems from the distinct roles played by macro and trace minerals in skeletal physiology. Calcium (Ca) and phosphorus (P) are the principal mineral components of bone and are required in relatively large amounts to ensure proper mineralization and structural integrity [28,29,30]. These macro minerals form the hydroxyapatite matrix, the primary structural component of bone. In contrast, trace minerals such as Zn, Mn, and Cu function mainly as enzyme cofactors involved in cartilage formation, collagen synthesis, and bone remodeling, and are therefore required in much smaller quantities [1,27,31].

Interestingly, tibia breaking strength at day 35 was significantly higher in the ILI group compared to the LLO and LLI groups (*p* < 0.05), suggesting a potential transient benefit of higher ITM supplementation on bone robustness under certain conditions. This may be attributed to greater mineral availability or enhanced enzymatic support during rapid growth. However, the absence of consistent differences across other bone parameters and time points suggests that this effect is likely marginal or context-dependent.

Overall, broilers receiving the low levels of OTMs exhibited bone development comparable to those receiving full or reduced levels of ITMs. This supports the idea that OTMs, due to their superior bioavailability and gastrointestinal stability, can fulfill physiological mineral requirements at lower inclusion levels [19,26]. While macro minerals remain the primary determinants of bone development, trace minerals appear to play a supportive role that can be met even at reduced levels, particularly when supplied in highly bioavailable organic forms. These results reinforce the feasibility of using OTMs at lower inclusion rates without impairing bone integrity, aligning with the principles of precision nutrition and environmentally sustainable feeding practices.

### 4.4. Oxidation Stress and Immunity

The results indicated that the source and level of trace minerals had no significant effect (*p* > 0.05) on oxidative stress or immunity. These findings are consistent with those of Vieira et al. [16], who similarly reported no significant differences in MDA concentrations in breast muscle and SOD activity in cardiac tissue of broilers fed varying levels and sources of trace minerals.

Lipid oxidation in muscle tissue, primarily assessed through MDA levels, serves as a key marker of oxidative stress and meat quality deterioration. SOD, an antioxidant enzyme that neutralizes superoxide radicals, is an important indicator of antioxidant defense mechanisms. The lack of variation in these parameters suggests that both OTMs and ITMs, even at reduced inclusion levels, are sufficient to maintain redox homeostasis under standard production conditions. This is particularly relevant because trace minerals such as Zn, Cu, and Mn act as essential cofactors for antioxidant enzymes including SOD and catalase [2,9].

This study also found no significant differences in total IgG antibody levels among dietary treatments (*p* > 0.05, Table 5). These findings are in agreement with previous studies reporting that replacing ITMs with OTMs maintained an adequate immune response in broilers [20,21,32]. However, Jain et al. [33] observed that supplementation with organic Zn, Se, and Cr enhanced humoral immune response and upregulated chTLR4 gene expression in the bursa and spleen of broilers.

Importantly, this study used dietary trace mineral levels (Cu 4 mg/kg, Mn 30 mg/kg, Zn 30 mg/kg) that were lower than NRC (1994) [17] recommendations (Cu 8 mg/kg, Mn 60 mg/kg, Zn 40 mg/kg) and breed-specific guideline (Cu 16 mg/kg, Mn 120 mg/kg, Zn 120 mg/kg), yet no adverse effects on growth performance, carcass traits, oxidative stability, or immunity were observed. These findings support growing evidence that broiler mineral requirements may be overestimated, particularly when highly bioavailable organic forms are used. OTMs exhibit superior stability in the gastrointestinal tract and greater absorption efficiency than their inorganic counterparts [1,5], allowing for reduced dietary inclusion without compromising physiological functions.

Moreover, lowering dietary trace mineral inclusion has important implications for environmental sustainability. Excessive supplementation, especially with inorganic minerals, leads to increased fecal mineral excretion and contributes to soil and water contamination [19,26]. Using organic minerals at lower levels not only maintains broiler performance and meat quality but also reduces environmental risks associated with mineral waste.

In this study, the potential contribution of amino acids from the OTMs was considered negligible due to both the inherently low quantity of amino acids present in the chelated complexes and the relatively low inclusion rate of the premix (0.5%). Consequently, this factor was not accounted for during diet formulation. However, recent findings of Xiong et al. [34] suggest that the observed effects may result from the combined influence of trace minerals and the additional amino acids provided by the chelated carriers. Although the difference in total amino acid content is extremely small, it remains important to consider the amino acid balance between amino acid-chelated and inorganic trace minerals in future research.

## 5. Conclusions

This study demonstrates that the use of OTMs at levels lower than those of commercial ITMs does not compromise growth performance, carcass traits, bone characteristics, oxidative stress, or immune response in broiler chickens. During the starter phase (1–21 days), OTMs significantly improved FCR compared to both standard and reduced levels of ITMs, indicating superior bioavailability. While carcass traits such as filet and thigh yields were enhanced with OTMs, no significant differences were observed in bone parameters, oxidative stress markers, or humoral immunity across treatments. Notably, reducing ITMs to levels below current commercial standards also maintained broiler performance, suggesting that existing inclusion rates may exceed actual physiological requirements. These findings support the strategic use of OTMs to promote more sustainable poultry production by improving mineral utilization efficiency and reducing environmental mineral excretion, without compromising bird health or productivity.

## Figures and Tables

**Table 1:** Composition of the basal diets.

Ingredients (%)	Starter (1–21 Days)	Grower (22–35 Days)
Corn	48.35	54.99
Soybean meal (44%)	32.57	28.73
Full fat soybean	10.00	7.50
Soybean oil	4.80	4.69
Lysine	0.12	0.17
Methionine	0.25	0.30
Limestone	1.44	1.27
Mono-calcium phosphate (MCP)	1.47	1.34
Choline chloride (60%)	0.05	0.06
Salt	0.44	0.44
Premix *	0.50	0.50
Calculated nutrient analysis		
Metabolizable energy (ME, kcal/kg)	3100.00	3150.00
Crude protein (%)	22.00	20.00
Calcium (%)	0.90	0.80
Total phosphorus (%)	0.70	0.65
Available phosphorus (%)	0.46	0.42
Digestible lysine (%)	1.20	1.20
Digestible methionine (%)	0.56	0.61
Digestible methionine + cystine (%)	0.90	0.85
Digestible threonine (%)	0.78	0.70
Digestible valine (%)	0.96	0.87

* Composition per kg: Vit A 10,000 IU, Vit D3 3,000 IU, Vit E 50 IU, Vit K3 3 mg, Vit B1 4 mg, Vit B2 7.5 mg, Niacin (B3) 50 mg, pantothenic acid (B5) 15 mg, Vit B6 5 mg, Vit B12 25 µg, folic acid 1 mg, Biotin 100 µg; Control (ILI): Zn 100 ppm, Cu 15 ppm, Fe 100 ppm, Mn 80 ppm, Se 0.2 ppm, I 3 ppm; LLO: Zn 30 ppm, Cu 4 ppm, Fe 11 ppm, Mn 30 ppm, Se 0.225 ppm, I 3 ppm, Cr 0.15 ppm; LLI: Zn 30 ppm, Cu 4 ppm, Fe 11 ppm, Mn 30 ppm, Se 0.2 ppm, I 3 ppm.

**Table 2:** Influence of trace mineral sources and levels on growth performance of broiler.

Treatments	Starter Phase (d 1–21)					Grower Phase (d 22–35)				Overall Performance (d 1–35)			
	Int wt (g/bird)	BWG (g/bird)	FI (g/bird)	FCR	Mortal (%)	BWG (g/bird)	FI (g/bird)	FCR	Mortal (%)	BWG (g/bird)	FI (g/bird)	FCR	Mortal (%)
ILI	48.87	963.39	1216.20	1.26 ^a^	1.56	1289.65	2191.26	1.71	0.00	2253.04	3407.46	1.51	1.56
LLO	48.87	982.63	1204.37	1.23 ^b^	0.00	1316.58	2228.20	1.70	0.00	2299.21	3432.56	1.50	0.00
LLI	48.65	963.43	1208.24	1.26 ^a^	0.00	1329.03	2157.18	1.62	0.00	2292.46	3365.42	1.47	0.00
SEM	0.08	6.05	5.91	0.01	0.36	17.17	19.62	0.02	0.00	19.43	22.07	0.01	0.36
*p*-value	0.50	0.34	0.72	0.02	0.12	0.65	0.35	0.15	1.00	0.60	0.47	0.21	0.12

Commercial levels of ITMs (ILI); low levels of OTMs (LLO); low levels of ITMs (LLI); initial weight (Int wt); body weight gain (BWG); feed intake (FI); feed conversion ratio (FCR); mortality (Mortal). standard errors of means (SEM); *p*-Value = significance level (*p* < 0.05). ^a,b^ Means within the same column with different superscripts are significantly different (*p* < 0.05).

**Table 3:** Influence of trace mineral sources and levels on carcass characteristics and meat quality of broiler.

Treatments	Average Weight			Relative Weight of Primal Cut (% of LW)								Internal Organs (% of LW)			Meat Quality	
	LW (g/bird)	PW (g/bird)	CW (%)	Head Neck	Wing	Breast	Filet	Thigh	Drum Stick	Leg Feet	Ribs	Heart	Liver	Gizzard	Ult pH	DL (%)
ILI	2577.94	2322.47	90.07	5.19	7.35	18.58	3.45 ^b^	10.52 ^a^	9.05	3.51	18.18	0.48	2.28	1.44	5.95	1.40
LLO	2595.63	2337.88	90.11	5.59	7.94	19.42	3.86 ^a^	11.99 ^b^	9.13	3.65	16.56	0.49	2.25	1.42	5.96	1.37
LLI	2619.75	2357.56	89.98	5.56	7.68	17.71	3.52 ^b^	11.18 ^ab^	9.34	3.50	17.70	0.49	2.34	1.50	5.94	1.45
SEM	38.81	34.79	0.15	0.13	0.16	0.33	0.07	0.23	0.10	0.05	0.43	0.01	0.06	0.04	0.00	0.03
*p*-value	0.91	0.92	0.94	0.38	0.31	0.10	0.03	0.02	0.50	0.37	0.30	0.93	0.84	0.63	0.94	0.09

Commercial levels of ITMs (ILI); low levels of OTMs (LLO); low levels of ITMs (LLI); Live weight (LW); Plucked weight (PW); Carcass weight (CW); Ultimate pH (Ult pH); Drip loss (DL). Standard errors of means (SEM); *p*-Value = Significance level (*p* < 0.05). ^a,b^ Means within the same column with different superscripts are significantly different (*p* < 0.05)

**Table 4:** Influence of trace mineral sources and levels on bone characteristics of broiler.

Treatments	Day 21							Day 35						
	Fresh Weight (mg)	Ash (%)	Length (mm)	W:L	BS (N)	Small OD (mm)	Large OD (mm)	Fresh weight (mg)	Ash (%)	Length (mm)	W:L	BS (N)	Small OD (mm)	Large OD (mm)
ILI	5754.47	41.16	76.71	74.85	196.69	7.03	19.20	13,913.04	39.16	101.42	136.52	406.40 ^a^	9.03	26.18
LLO	6137.59	40.28	76.45	80.25	197.10	7.09	19.84	14,355.36	37.02	101.87	139.90	345.01 ^b^	8.78	26.15
LLI	6054.38	38.80	76.45	79.16	192.82	7.15	19.87	13,714.88	40.7	101.43	133.98	342.62 ^b^	8.98	25.66
SEM	113.69	0.51	0.38	1.27	4.06	0.07	0.14	368.55	0.84	1.17	2.46	11.00	0.11	0.20
*p*-value	0.37	0.16	0.96	0.19	0.9	0.82	0.10	0.78	0.21	0.99	0.63	0.02	0.64	0.49

Commercial levels of ITMs (ILI); low levels of OTMs (LLO); low levels of ITMs (LLI); Weight: length (W:L); Breaking strength (BS); Small outer diameter (small OD); Large outer diameter (Large OD). Standard errors of means (SEM); *p*-Value = Significance level (*p* < 0.05). ^a,b^ Means within the same column with different superscripts are significantly different (*p* < 0.05)

**Table 5:** Influence of trace mineral sources and levels on oxidative stress and humeral immunity of broiler.

Treatments	Day 21		Day 35		
	SOD (% Inhibition Rate)	MDA (nmol/mL)	SOD (% Inhibition Rate)	MDA (nmol/mL)	Total IgG (ng/mL)
ILI	65.76	6.39	61.94	7.88	103.51
LLO	66.00	6.15	65.62	7.52	106.40
LLI	58.76	6.34	67.68	6.77	100.09
SEM	0.42	0.10	0.59	0.08	1.23
*p*-value	0.61	0.31	0.99	0.70	0.91

Commercial levels of ITMs (ILI); low levels of OTMs (LLO); low levels of ITMs (LLI); Superoxide dismutase activity (SOD); Malondialdehyde (MDA); Total IgG antibodies (Total IgG). Standard errors of means (SEM); *p*-Value = Significance level (*p* < 0.05).

## Data Availability

Data are available from the corresponding author upon request.
